# Agricultural Capacity to Increase the Production of Select Fruits and Vegetables in the US: A Geospatial Modeling Analysis

**DOI:** 10.3390/ijerph14101106

**Published:** 2017-09-23

**Authors:** Zach Conrad, Christian J. Peters, Kenneth Chui, Lisa Jahns, Timothy S. Griffin

**Affiliations:** 1 Grand Forks Human Nutrition Research Center, US Department of Agriculture, Agricultural Research Service, 2420 2nd Avenue N, Grand Forks, ND 58203, USA; lisa.jahns@ars.usda.gov; 2 Friedman School of Nutrition Science and Policy, Tufts University, 150 Harrison Ave. Boston, MA 02111, USA; christian.peters@tufts.edu (C.J.P.); timothy.griffin@tufts.edu (T.S.G.); 3 School of Medicine, Tufts University, 136 Harrison Ave. Boston, MA 02111, USA; kenneth.chui@tufts.edu

**Keywords:** fruits and vegetables, GIS, model, geospatial, capacity

## Abstract

The capacity of US agriculture to increase the output of specific foods to accommodate increased demand is not well documented. This research uses geospatial modeling to examine the capacity of the US agricultural landbase to increase the per capita availability of an example set of nutrient-dense fruits and vegetables. These fruits and vegetables were selected based on nutrient content and an increasing trend of domestic production and consumption. Geographic information system models were parameterized to identify agricultural land areas meeting crop-specific growing requirements for monthly precipitation and temperature; soil depth and type; cropland availability; and proximity to existing production centers. The results of these analyses demonstrate that crop production can be expanded by nearly 144,000 ha within existing national production centers, generating an additional 0.05 cup-equivalents of fruits and vegetables per capita per day, representing a 1.7% increase above current total F&V availability. Expanding the size of national crop production centers can further increase the availability of all F&V by 2.5%–5.4%, which is still less than the recommended amount. Challenges to increasing F&V production in the US include lack of labor availability, barriers to adoption among producers, and threats to crop yields from environmental concerns.

## 1. Introduction

American consumers depend almost entirely on imports for some of their favorite foods, like cocoa and coffee [1,2], yet despite the growing importance of international food markets, the US still produces most of the food that the population consumes [1,3]. Therefore, increased consumer demand for many types of food, particularly perishables, would likely spur increased domestic production of agricultural goods. However, the capacity of US agriculture to increase the output of specific foods to accommodate increased demand is not well documented.

One way that shifts in consumer food demand may occur is if Americans improved their diet quality. Adequate fruit and vegetable (F&V) consumption is now a mainstay of the Dietary Guidelines for Americans [4] because of the relationship between regular consumption and the reduced risk of chronic disease [5]. Despite routine public health messaging and an ongoing national campaign [6] to increase F&V consumption, there has not been a meaningful improvement in consumption patterns for decades [7]. Yet, although Americans continue to fall far short of meeting recommended daily F&V consumption amounts [8], modest increases have recently been documented [8,9].

If Americans consumed more F&V, domestic production would likely increase to some degree because nearly one-half of the fruits and two-thirds of the vegetables consumed in the US are produced domestically [1,3]. While yield improvements from technological innovations (e.g., improved disease and pest resistance and improved fruit set) and the adoption of intensive management practices (e.g., high density planting and global positioning systems) are largely responsible for recent increases in F&V production, the viability of these approaches is dependent on the availability of suitable agricultural land [10,11]. Because many F&V crops require highly specific growing conditions, identifying suitable agricultural sites is of primary importance to increasing production.

A growing body of research has examined the connection between F&V demand (actual and potential), agricultural land use, and food production at different spatial scales. Giombolini et al. [12] found that the Willamette Valley in Oregon produced enough food to meet approximately 20%–30% and 10%–30% of the Willamette Valley population’s fruit and vegetable recommendations, respectively, as part of a complete diet. Kremer and Shrueder [13] estimated that the Philadelphia foodshed (which represents 37 counties around Philadelphia that have farms that supply food to the city’s residents) produces more than enough F&V to meet the current and recommended consumption amounts of the city’s residents. In Detroit (MI), Colasanti and Hamm [14] found that vacant land areas could produce enough F&V to meet 42% and 76% of current fresh fruit and vegetable consumption, respectively, of the city’s residents, using food storage and season extension technologies. Peters et al. [15] estimated that if the share of the population in New York State consuming local food increased, it was more likely that land in the state would be allocated to higher value crops like F&V at the expense of grains and dairy. At the national level, Young and Kantor [16] estimated that an additional 5–7 million acres (2–2.8 million hectares) of land would be need to be cultivated in order for the population to meet F&V consumption recommendations; later, Buzby et al. [17] updated this estimate to 13 million acres (5.3 million hectares). These studies present useful methodologies for estimating the agricultural capacity to increase the production of F&V for the general population at different spatial scales, but there remains is limited information on the agricultural capacity to increase the production of specific F&V that provide nutrients of public health concern. This research is needed to identify specific sites for viable crop production, and to better understand how crop growth requirements and geospatial land use constraints (i.e., availability of zoned agricultural land not currently cultivated) may impact the availability of food and nutrients for consumers.

The goal of this study was to examine the capacity of the US agricultural land base to increase the per capita availability of selected F&V. We chose a specific set of F&V as an example based on their contribution of nutrients of public health importance. Our approach is innovative in that it uses a series of geospatial models that are parameterized for precipitation, temperature, soil depth, soil type, and land availability. In addition, we recognize that supply chain infrastructure is needed to accommodate increased agricultural production, so we focus on areas in and around current production centers because these co-locate with the requisite supply chain infrastructure.

## 2. Materials and Methods

### 2.1. Identifying Nutrient Dense Fruits and Vegetables

The F&V used in this example were identified using an indexing method that computed a nutrient density score for each of 407 F&V [18] on the basis of fiber, calcium, magnesium, and potassium, all nutrients of public health concern [19], per serving. In order to represent F&V most likely to be adopted by consumers and agricultural producers, F&V were further restricted to those with positive trends of consumption [20] and production [21] over the most recent five year period, based on the slope of a simple regression equation that plotted consumption or production amount against time. F&V were further categorized by MyPlate subgroups [22]. In total, two fruits (dates and kiwis) and five vegetables (broccoli, lima beans, sweet potatoes, Great Northern beans, and mushrooms) were included in this analysis.

### 2.2. Identifying Production Centers

The complex and specialized nature of F&V supply chains means that supply chain enterprises co-locate with areas of high agricultural production [23] (hereafter, production centers).We therefore recognize that increased cultivation of F&V is most likely to occur near current production centers, so we focused our analysis on these areas. For each crop included in this analysis, we identified production centers as counties that represented at least 10% of mean (2002–2012) national acreage [21]. In cases where eligible counties abutted one another, the counties were considered to be a single production center. The seven crops included in this analysis were associated with 11 national production centers located in six states (Table 1).

### 2.3. Geospatial Modeling

For each F&V in each production center, a site suitability geographic information system (GIS) model was created to identify suitable land areas for crop production in and around each production center (ArcGIS 10.1, Esri, Redlands, CA, USA). Site suitability analyses use crop growing requirements and characteristics of a given land parcel to predict future suitability for crop production in that locale [24]. A key feature of a geospatial site suitability model is the ability to visually overlay multiple maps, with each map depicting a distinct geospatial parameter such as precipitation, temperature, and soil characteristics. A site suitability model allows the user to identify specific locations that simultaneously meet pre-specified conditions for each parameter. For each crop model, data were mapped using a State Plane North American Datum 1983 projected coordinate system that was specific to the locale of each production center. All data were analyzed in raster format with a resolution of 30 m × 30 m.

Land areas were considered to be suitable for the production of a given crop if all of the crop-specific growing conditions were satisfied. Growing conditions for each crop were identified through published reports [25,26,27,28,29,30,31,32,33,34] and subsequently verified by personal communication with farm advisors and plant scientists from the USDA Cooperative Extension System. The following growing conditions were included in this analysis: mean monthly (1981–2010) precipitation and mean monthly (1981–2010) surface temperature (minimum and maximum) were collected from the Parameter-elevation Regressions on Independent Slopes Model, maintained by the PRISM Climate Group at Orgon State University [35,36]; soil depth (minimum) and texture class (particle size) were collected from the Gridded Soil Survey Geographic database, maintained by US Department of Agriculture (USDA) Natural Resources Conservation Service [37]; and land use (fallow agricultural land) was collected from the Cropland Data Layer, maintained by USDA National Agricultural Statistics Service [38]. Only the growing conditions relevant to a given crop were included in a given crop model (Supplementary Tables S1–S10).

In cases where geospatial data lacked sufficient resolution to be parameterized according to the information provided by published reports and personal communication with experts, the range of values for the production center of a given crop were used to represent the growing conditions for that crop. For example, monthly lower and upper temperature bounds for each crop in each production center were established by adopting the monthly minimum and maximum temperature readings, respectively, in that production center. In cases where high resolution geospatial data on crop-specific land use were available [38], we identified clusters of land within counties in which at least 95% of a given crop was cultivated, and used these clusters to establish the monthly lower and upper temperature bounds for that crop.

### 2.4. Modeling Scenarios

In order to examine the potential to increase food production under scenarios of increased cultivated land area, we constructed a series of buffer areas around each production center that extended 5, 10, 15 and 20 km from all sides of each production center (in other words, increasing the area while maintaining the shape). This resulted in five distinct scenarios: (1) no cropland expansion, (2) 5 km expansion, (3) 10 km expansion, (4) 15 km expansion, and (5) 20 km expansion.

### 2.5. Estimating Current and Potential Food Availability

State-level crop yield data were collected from USDA Census of Agriculture [21]. Yield data were applied to the land area data generated from the site suitability analysis in order to estimate potential production in and around each production center, on a farm-weight basis. These data were then adjusted for food losses and waste that occur as food moves through the supply chain [20], and were converted to cup-equivalents using the conversion factors provided by the USDA Loss-adjusted Food Availability (LAFA) data series [20]. For the purposes of this analysis, we assumed that all potential food availability would be equally distributed amongst all Americans. Therefore, data on total food availability were divided by the total US population [39] in order to estimate per capita consumption.

## 3. Results

**Figure 1:**
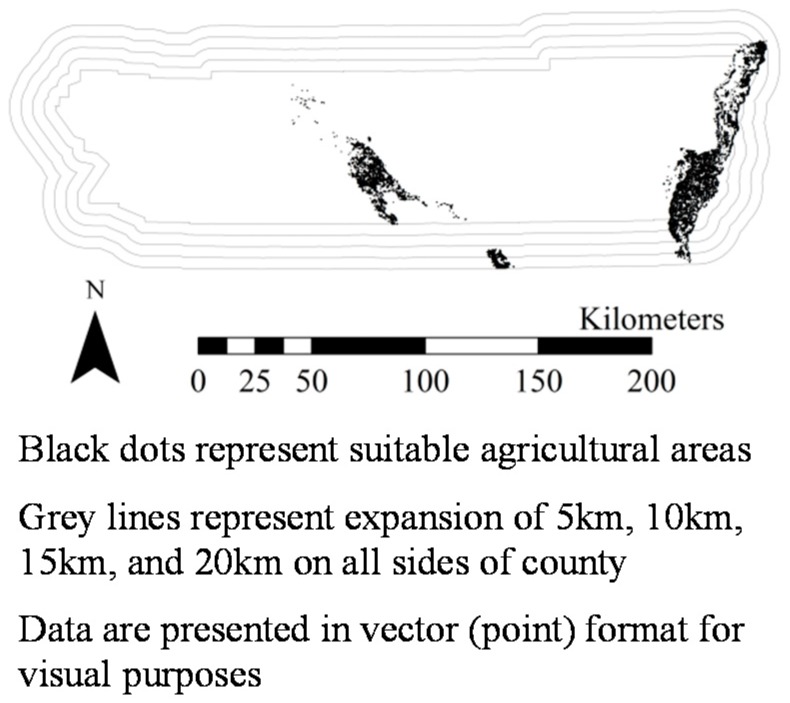
Suitable agricultural land for dates in Riverside County, California.

**Figure 2:**
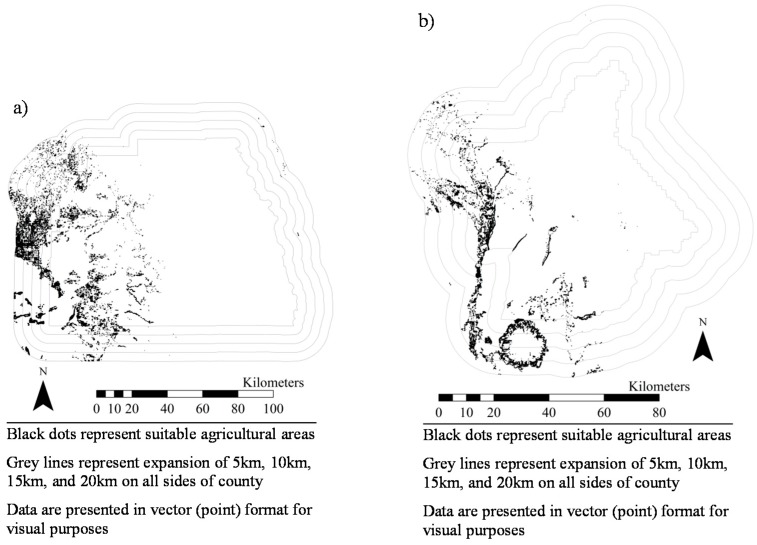
Suitable agricultural land for kiwis in (**a**) Tulare County, California and (**b**) Butte County, California.

**Figure 3:**
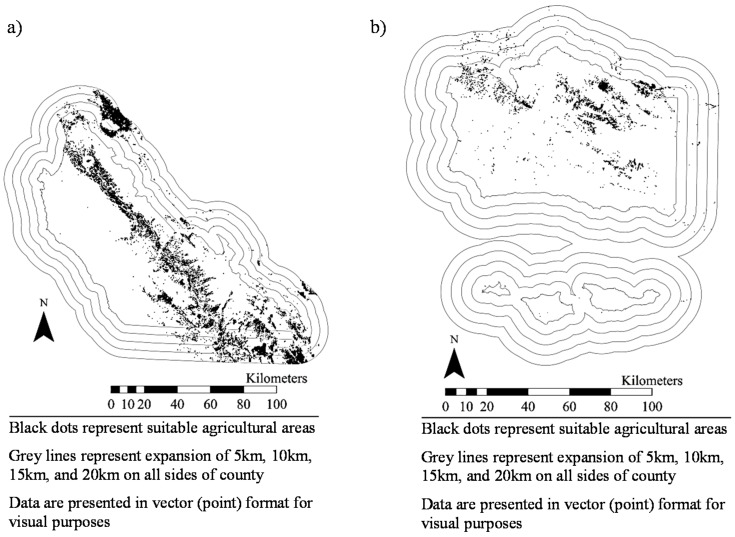
Suitable agricultural land for broccoli in (**a**) Monterey County, California and (**b**) Santa Barbara County, California.

**Figure 4:**
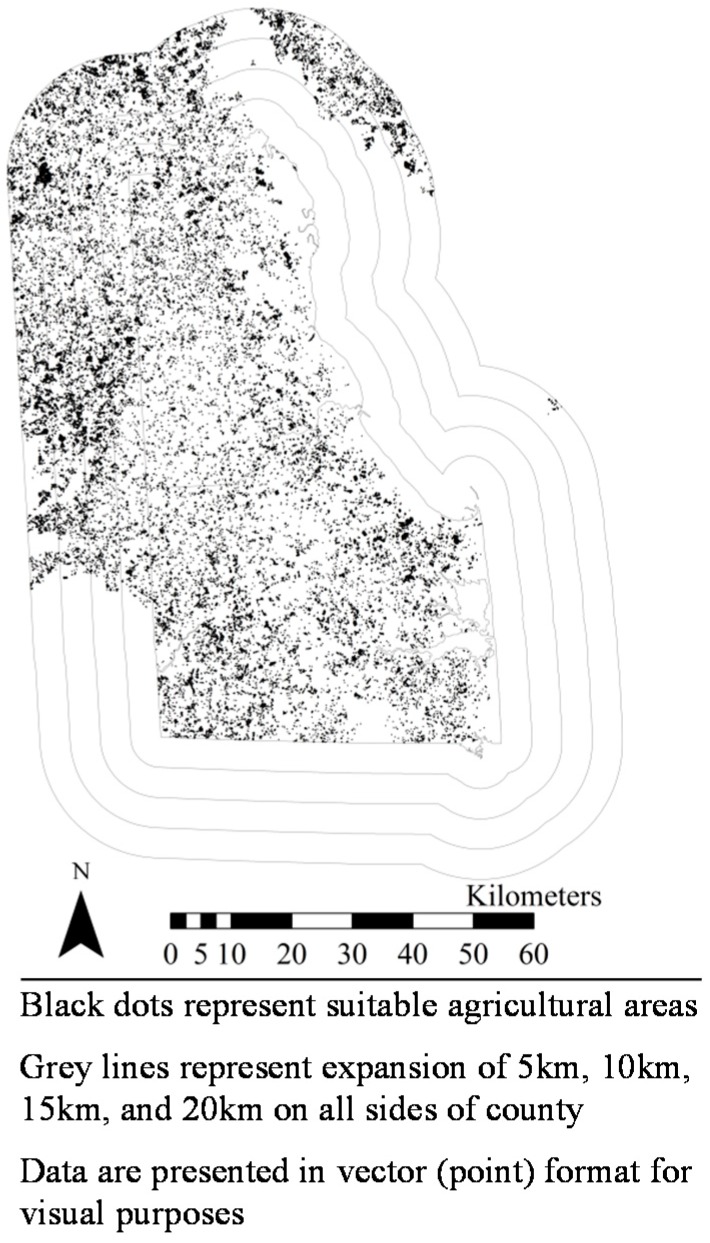
Suitable agricultural land for lima beans in Sussex and Kent Counties, Delaware.

**Figure 5:**
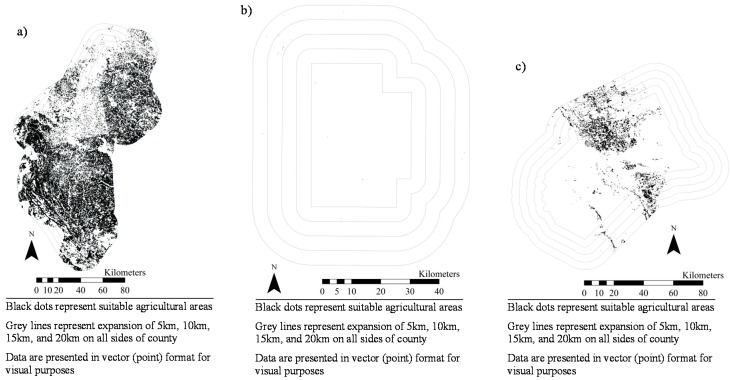
Suitable agricultural land for sweet potatoes in (**a**) Johnston, Nash, Sampson, and Wilson Counties, North Carolina, (**b**) Calhoun County, Mississippi, and (**c**) Merced County, California. Suitable land area for sweet potato production in Calhoun County, Mississippi were identified in this geospatial analysis, but are not clearly visible in Figure 5b due to the small land areas.

**Figure 6:**
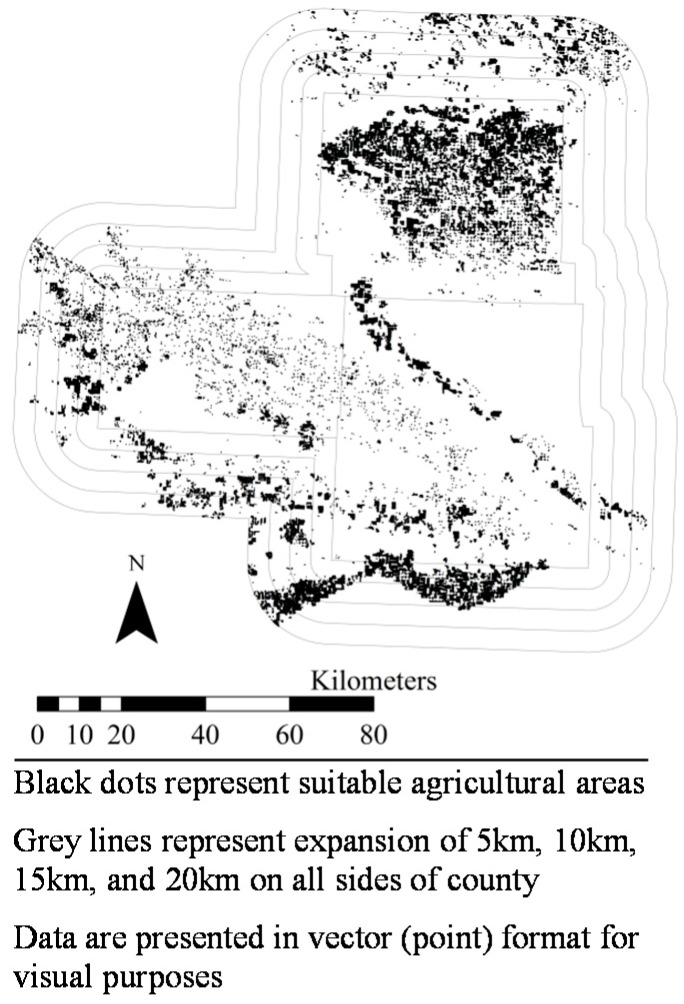
Suitable agricultural land for Great Northern beans in Scotts Bluff, Box Butte, and Morrill Counties, Nebraska.

**Figure 7:**
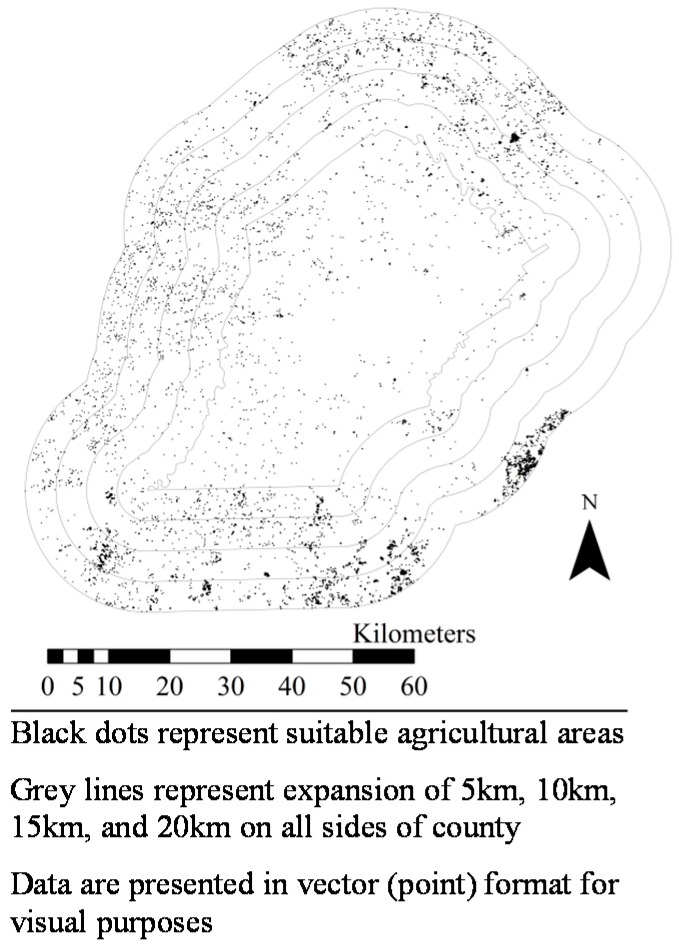
Suitable agricultural land for mushrooms (agaricus) in Chester County, Pennsylvania.

Figure 1, Figure 2, Figure 3, Figure 4, Figure 5, Figure 6 and Figure 7 display the geospatial results of the site suitability models, and Table 2 displays the tabular results. For each crop, additional suitable cropland was identified within the existing production centers, and the amount of suitable land increased with every 5 km expansion of the production centers. In total, nearly 144,000 ha of suitable land was identified for increased production of dates, kiwis, broccoli, lima beans, sweet potatoes, Great Northern beans, and mushrooms within existing production centers. This increased by 210,000 ha, 281,000 ha, 345,000 ha, and 412,000 ha when production centers were expanded by 5, 10, 15, and 20 km, respectively.

Table 3 displays the daily per capita availability of dates, kiwis, broccoli, lima beans, sweet potatoes, Great Northern beans, and mushrooms if suitable agricultural land circumjacent to each production center were brought into production. Approximately 0.04 additional cups of F&V could be made available to each individual in the US every day without any expansion of existing production centers. This increased by 0.06 cups, 0.09 cups, 0.11 cups, and 0.14 cups per person per day when production centers expanded by 5 km, 10 km, 15 km, and 20 km, respectively.

Nearly all Agaricus mushrooms produced in Chester County (PA) are grown in indoor, climate-controlled environments, thus limiting the parameters used in the geospatial analysis for this crop compared to other crops which are produced in the open. Post hoc sensitivity analyses were therefore conducted by omitting mushrooms from estimates of total suitable agricultural land area and per capita availability of nutrient dense fruits and vegetables. Omitting mushrooms from these analyses reduced total agricultural land area estimates by <1% for each modeling scenario, and attenuated per capita fruit and vegetable availability estimates by 3%–14% for each modeling scenario (data not shown).

## 4. Discussion

In this national geospatial analysis, we identified enough suitable cropland within existing F&V production centers to moderately increase the daily per capita availability of select F&V in the US. We also identified additional suitable cropland with every 5 km expansion of the production centers. Within existing production centers a total of nearly 144 thousand additional ha of cropland could be brought into production, resulting in an additional 0.04 cup-equivalents of F&V per capita per day. This represents a 1.7% increase over the 2.6 total daily per-capita cup equivalents of all F&V consumed by individuals in the US (Conrad et al., in press) [40] and expanding the production centers of these crops by 5–20 km could increase the daily per capita availability of F&V by 0.06–0.14 cups per day, representing an increase of 2.5%–5.4% over current total F&V availability.

A growing body of research has examined the connection between F&V demand (actual and potential), agricultural land use, and food production at different spatial scales [12,13,14,15,41]. At the national level, Young and Kantor [16] estimated that an additional 5–7 million acres (2–2.8 million hectares) of land would need to be cultivated in order for the population to meet F&V consumption recommendations; later, Buzby et al. [17] updated this estimate to 13 million acres (5.3 million hectares). While our results demonstrate that expansion of some current F&V production centers can moderately increase the total per capita availability of F&V, substantial increases in production would still be needed in order to accommodate population-wide adoption of dietary recommendations for F&V.

Additional production of F&V in the US could be achieved by further expanding the size of the production centers, increasing the number of production centers, converting land currently used for crops like grains and oilseeds to F&V production, and technological innovations to increase F&V crop yields. Indeed, others have demonstrated the potential for agricultural land to be reallocated to higher value crops like F&V at the expense of grains and dairy, based on different land use values across distinct land parcels suitable for the production of distinct crops [15]. Additionally, suitable land areas likely exist for crops other than those included in this analysis, so further increases in F&V production could be achieved by cultivating this land. Yet several factors present challenges to these approaches, such as labor availability, barriers to adoption among producers, and threats to crop yields from environmental concerns.

Access to labor is particularly problematic for F&V operations. The future availability of farmworkers is uncertain given the decline in the number of seasonal, short-term workers since the late 1990s [42], and the declining number of young farm operators entering the agricultural sector [43]. Many F&V crops require specialized knowledge, planning, and management from farm operators in order to ensure adequate production, and the time needed to develop this knowledge and implement these practices could be a barrier to adoption for non-F&V producers [44]. Although mechanization is more common for vegetables than fruit, and for processed produce than for fresh varieties, even crops grown under a mechanized production system may still require field grading and packing by hand [45] and machines cannot replicate the dexterity of farmworkers, which is needed to prevent crop damage that can reduce the market value of harvested crops, particularly those destined for the fresh market [45].

Changes in environmental conditions will also influence the viability of the F&V sector. For example, multi-year drought conditions have reduced water availability in California, threatening the vitality of the largest F&V industry in the country [46]. Reduced crop yields were reported on nearly 1.2 million acres (0.49 million hectares) in 2013 due to a shortage of surface and ground water [47], and aquifer recharge could take many years to reach pre-drought levels [48]. The prospect for increasing F&V production in California is made even more uncertain by the effects of salinization of soil and water sources [49,50], as well as the effects of climate change projections that include altered patterns of precipitation and temperature [51,52,53], all of which have the potential to reduce crop yields [54,55,56].

Even if the challenges associated with increasing domestic production were overcome, imports of F&V would still be essential for meeting increased consumer demand. Imports are needed to smooth out strong seasonal price fluctuations that often result from climatic seasonality or unexpected weather events [57,58]. In cases where the availability of domestically produced F&V lags behind demand (as a result of the time it takes to establish productive orchards and increase labor availability), imported F&V may be able to fill this gap. Indeed, F&V imports have increased steadily since 1975 [59,60].

There are several key strengths to this study. We included a broad range of parameters that allowed for a robust analysis of site suitability. Our models were specific to each crop, which allowed for a higher level of specificity when establishing the upper and lower bounds of the relevant growing conditions than would have been possible if our crop focus was more broadly defined (for example, field crops vs. specialty crops). By narrowing our focus to crops with increasing trends of consumption and domestic production, we were able to identify, based on our assumptions, crops that would be most likely to be adopted by consumers and producers. Finally, we focused our analyses on areas where supply chain infrastructure was likely to be present in order to address the reality that agricultural production requires the co-location of supply chain infrastructure in order for food to reach consumers.

This study has several limitations. It has been reported that recent drought conditions in California have encouraged producers to fallow at least 410,000 acres (166,000 hectares) of cropland [61]. This land could have been identified as suitable cropland by the geospatial models we used in this study, and may have contributed to an overestimation of suitable land area for crops with production centers in California. Data on irrigation limitations were not included due to lack of data availability, so we assessed whether crop-specific water needs were met based on precipitation, which would have resulted in a conservative estimate of suitable land area. This study may not have included all of the production centers for each crop because of limited data availability for some crops in some areas. Incomplete data on the location of national production centers would likely have contributed to an underestimation of suitable land area for some crops. We chose a sample of seven F&V based upon content of specific nutrients, but other F&V also contribute valuable nutrients. Mushrooms, in particular, pose challenges for geospatial analyses because most are produced under climate-controlled production systems, so standard parameters such as precipitation, surface temperature, soil depth, and soil texture class are not relevant. Future research should identify other relevant parameters for mushroom production, such as spatial availability of critical inputs such as substrate (e.g., straw, manure, and peat moss), and explore methods for accurate parameterization in geospatial analyses. Finally, increased supply of F&V may not be distributed equally among all individuals in the US, given limited access to nutritious and affordable food in some communities [62].

Additional research is needed to examine the availability of suitable agricultural land in and around smaller production centers throughout the country in order to fully capture the national potential to increase F&V production. These site suitability analyses could be integrated into existing modeling frameworks [63,64,65] that have been used to examine the capacity of supply chain infrastructure to handle additional volume. This research should focus on the potential to increase production in areas least likely to experience environmental degradation and natural resource limitations.

## 5. Conclusions

In this geospatial modeling study we show that there is enough suitable agricultural land in and around existing production centers to increase the availability of certain nutrient dense F&V in the US. Yet even greater production of F&V would be needed in order to accommodate increased adherence to dietary guidance, and increased imports will likely be needed. Challenges to increasing F&V production in the US include lack of labor availability, barriers to adoption among producers, and threats to crop yields from environmental concerns. Further research is needed to examine the potential to increase production for a broader range of crops in areas least likely to experience environmental degradation and natural resource limitations.

## Figures and Tables

**Table 1:** National production centers of nutrient dense fruits and vegetables.

Crop	MyPlate Subgroup	Production Center		
		County	State	Proportion of National Acreage (%)
**Fruits**				
Dates	n/a	Riverside	California	55
Kiwis	n/a	Tulare	California	35
		Butte	California	24
**Vegetables**				
Broccoli	Dark green	Monterey	California	39
		Santa Barbara	California	20
Lima beans	Starchy	Sussex and Kent	Delaware	25
Sweet potatoes	Red and orange	Johnston, Nash, Sampson, and Wilson	North Carolina	27
		Calhoun	Mississippi	12
		Merced	California	11
Great Northern beans	Beans and peas	Scotts Bluff, Box Butte, and Morrill	Nebraska	70
Mushrooms, Agaricus	Other	Chester	Pennsylvania	29

**Table 2:** Current agricultural land area and potential agricultural land area suitable for the production of nutrient dense fruits and vegetables.

Crop	Currently Harvested ^1^	Production Center Expansion ^2^				
		No Expansion	5 km	10 km	15 km	20 km
	10^3^ ha ^3^					
Total	148.7	143.9	209.5	280.7	344.7	412.2
Dates	3.0	6.0	7.3	8.6	9.8	10.3
Kiwis	1.7	10.1	14.5	19.4	24.6	30.5
Broccoli	54.1	10.7	12.1	14.7	21.1	27.6
Lima beans	19.2	3.8	4.3	5.1	6.3	8.1
Sweet potatoes	43.6	82.7	133.9	188.7	235.3	284.5
Great Northern beans	26.6	30.6	37.1	43.8	46.8	49.7
Mushrooms, Agaricus	0.4	0.1	0.2	0.4	0.8	1.4

Notes: **^1^** National mean (2002–2012), Census of Agriculture. **^2^** Represents distance from all sides of production center. **^3^** Values are cumulative.

**Table 3:** Current and potential per capita availability of nutrient dense fruits and vegetables.

Crop	Current ^1^	Production center expansion ^2^				
		No Expansion	5 km	10 km	15 km	20 km
	10^−3^ cup eq. per person per day ^3,4^					
Total	84.9	43.6	63.5	86.3	110.1	138.0
Dates	2.4	3.4	4.1	4.8	5.5	5.8
Kiwis	1.6	2.7	3.8	5.1	6.5	8.0
Broccoli	42.8	6.0	6.8	8.2	11.8	15.4
Lima beans	<0.02	0.2	0.2	0.3	0.3	0.4
Sweet potatoes	11.7	23.0	37.0	51.9	64.6	78.0
Great Northern beans	5.9	7.2	8.7	10.3	11.0	11.7
Mushrooms, Agaricus	20.6	1.2	2.9	5.6	10.3	18.6

Notes: **^1^** National mean (2007–2011), Loss-Adjusted Food Availability (LAFA) data series. **^2^** Represents distance from all sides of production center. **^3^** Adjusted for losses at the farm, retail, and consumer levels. **^4^** Values are cumulative.

## Supplementary Materials

The following are available online at www.mdpi.com//1660-4601/14/10/1106/s1, Table S1: Growing conditions for dates in Riverside County, California, by month; Table S2: Growing conditions for kiwis in Tulare County, California, by month; Table S3: Growing conditions for kiwis in Butte County, California, by month; Table S4: Growing conditions for broccoli in Monterey County, California, by month; Table S5: Growing conditions for broccoli in Santa Barbara County, California, by month; Table S6: Growing conditions for lima beans in Sussex and Kent Counties, Delaware, by month; Table S7: Growing conditions for sweet potatoes in Johnson, Nash, Sampson, and Wilson Counties, North Carolina, by month; Table S8: Growing conditions for sweet potatoes in Calhoun County, Mississippi, by month; Table S9: Growing conditions for sweet potatoes in Merced County, California, by month; Table S10: Growing conditions for Great Northern beans in Scotts Bluff, Box Butte, and Morrill Counties, Nebraska, by month.
